# Assessment of Artificial Intelligence Automatic Multiple Sclerosis Lesion Delineation Tool for Clinical Use

**DOI:** 10.1007/s00062-021-01089-z

**Published:** 2021-09-20

**Authors:** Amalie Monberg Hindsholm, Stig Præstekjær Cramer, Helle Juhl Simonsen, Jette Lautrup Frederiksen, Flemming Andersen, Liselotte Højgaard, Claes Nøhr Ladefoged, Ulrich Lindberg

**Affiliations:** 1 Department of Clinical Physiology, Nuclear Medicine and PET, Rigshospitalet, University of Copenhagen, Blegdamsvej 9, 2100 Copenhagen east, Denmark; 2 Danish Multiple Sclerosis Center, Department of Neurology, Rigshospitalet, University of Copenhagen, Valdemar Hansens Vej 13, 2600 Glostrup, Denmark

**Keywords:** Magnetic resonance imaging, Convolutional neural network, Clinical implementation, White matter hyperintensity

## Abstract

**Purpose:**

To implement and validate an existing algorithm for automatic delineation of white matter lesions on magnetic resonance imaging (MRI) in patients with multiple sclerosis (MS) on a local single-center dataset.

**Methods:**

We implemented a white matter hyperintensity segmentation model, based on a 2D convolutional neural network, using the conventional T2-weighted fluid attenuated inversion recovery (FLAIR) MRI sequence as input. The model was adapted for delineation of MS lesions by further training on a local dataset of 93 MS patients with a total of 3040 lesions. A quantitative evaluation was performed on ten test patients, in which model-generated masks were compared to manually delineated masks from two expert delineators. A subsequent qualitative evaluation of the implemented model was performed by two expert delineators, in which generated delineation masks on a clinical dataset of 53 patients were rated acceptable (< 10% errors) or unacceptable (> 10% errors) based on the total number of true lesions.

**Results:**

The quantitative evaluation resulted in an average accuracy score (F1) of 0.71, recall of 0.77 and dice similarity coefficient of 0.62. Our implemented model obtained the highest scores in all three metrics, when compared to three out of the box lesion segmentation models. In the clinical evaluation an average of 94% of our 53 model-generated masks were rated acceptable.

**Conclusion:**

After adaptation to our local dataset, the implemented segmentation model was able to delineate MS lesions with a high clinical value as rated by delineation experts while outperforming popular out of the box applications. This serves as a promising step towards implementation of automatic lesion delineation in our MS clinic.

**Supplementary Information:**

The online version of this article (10.1007/s00062-021-01089-z) contains supplementary material, which is available to authorized users.

## Introduction

Multiple sclerosis (MS) is a neuroinflammatory disease of the central nervous system (CNS) affecting more than 2.3 million people worldwide. The disease manifests as areas of neuronal demyelination, causing axonal injury and loss, which is visible as hyperintense lesions on T2-weighted magnetic resonance images (MRI). Registration of these lesions is today an important part of diagnosis and monitoring of the disease. During diagnosis, lesion dissemination in time and space is qualitatively determined as part of the McDonald criteria, while lesion growth and the disappearance or appearance of new lesions is monitored during follow-up examinations to assess disease development [1]. Full delineation of MS lesions is not feasible in clinical routine and is thus not part of the assessment [2]. In recent years MR biomarkers, such as the total lesion load of a patient (e.g., number and volume of lesions) has proved prognostic for long-term disability and can thus be used to stratify MS patients [3–6], increasing the need for MS delineation as part of clinical routine.

Manual lesion delineation is a difficult and laborious process, prone to high interrater and intrarater variability due to the heterogeneous nature of MS lesions [7, 8]. To this end, fully automatic lesion delineation models have been researched for many years [9]. Recently, models based on a subgroup of machine learning called deep learning have dominated the field, with models based on convolutional neural networks (CNN) massively represented in the top tier of all international MS lesion segmentation challenges [8, 10, 11]. The best performing methods evaluated on various MS challenge datasets [12–18] achieve mean dice scores in the range of 0.52–0.68 [9].

Only some of these methods have been evaluated on local datasets, which often include MRI protocols, scanner types and MS diagnoses not well-represented in the challenge datasets. This poses a challenge, since deep learning methods tend to not generalize well across domains, preventing currently available deep learning techniques from translation into clinical practice. One strategy to increase generalization is to apply transfer learning of the original model to a subset of the patients in the local dataset, as demonstrated by Ghafoorian et al. who achieved a dice score of 0.76 on the local dataset, compared to 0.05 when not using any transfer learning [19]. Valverde et al. proposed transfer learning to local unseen data by updating only the weights in the last three fully connected layers of their deep learning framework, denoted nicMSlesions, which was originally trained on two public challenge datasets [20]. They found that retraining on as little as one patient could improve segmentation performance between scanner domains and reported a dice score of 0.52 when tested on a clinical dataset and retrained on a subset of 30 patients. While direct comparison of such studies is not straightforward as the performance is highly dependent on the evaluation cohort, it is apparent that retraining the models trained on challenge datasets is a requirement for acceptable MS lesion delineation.

We aimed at the introduction and validation of an existing pretrained algorithm for automated white matter hyperintensity (WMH) segmentation on our local clinical dataset acquired with a single scanner. The dataset employed in this study consisted of patients with a high ratio of dirty white matter and includes stages of disease progression which are generally not well-represented in challenge datasets. We adapted the algorithm through transfer learning and parameter optimization for optimal performance on the local dataset consisting of patients with early signs of MS as well as confirmed MS. Two experienced delineators examined the clinical value of the automatic delineations to assess feasibility of automatic lesion delineation in clinical use.

## Material and Methods

### Subjects

The dataset of this study is comprised of data from two separate clinical research studies and one retrospective collection of patients from routine MS investigations. The two clinical studies were not directed at optimizing lesion segmentation. The first research study, RS1 (protocol no. H‑1-2014-132) investigated changes in the permeability of the blood brain barrier (BBB) before and after treatment with one of the two types of second line medication: natalizumab and fingolimod. We included 50 patients diagnosed with relapsing remitting MS (RRMS) enrolled between April 2015 and April 2018, from the ongoing study. The second study, RS2 (protocol no H‑D-2008-002), was published in Brain by Cramer et al. in 2015 [21] and investigated the ability to predict conversion from optic neuritis (ON) to MS by the permeability of the BBB. A total of 43 patients enrolled in the study between June 2011 and December 2012 and diagnosed with ON, were included in our analysis. Both studies were single center single scanner studies, performed at the same department, and both studies involved manually drawn WM lesion masks, which were also used in this study. The retrospective clinical dataset consisted of patients diagnosed with RRMS who were referred for a clinical MRI evaluation at the same department as the two clinical studies. We randomly selected 53 consecutive patients examined between February and May 2019 from a larger collection. Scanner parameters for all three datasets are presented in Sect. “MR Imaging Protocol”. All RRMS patients were diagnosed according to the revised McDonald criteria from 2017 [1].

Table 1 presents the demographic characteristics of the three datasets. No score on the expanded disability status scale (EDSS) was estimated regarding the patients from RS2. The EDSS information of the clinical dataset was not disclosed.

**Table 1:** Summary of patient demographics

Patient characteristics	RS1	RS2	Clinical
*No. of patients*	50	43	53
*Sex (% female)*	83%	77%	72%
*Mean age in years (range)*	29 (24–39)	36 (29–46)	45 (22–77)
*Mean EDSS*	1.6	–	–
*Diagnosis*	RRMS	ON	RRMS

*EDSS* expanded disability status scale, *ON* optic neuritis, *RRMS* relapsing remitting multiple sclerosis

#### MR Imaging Protocol

Acquisition parameters of the three datasets are presented in Table 2. The same scanner and protocol were used for acquiring RS1 and the clinical dataset. RS2 was acquired using a different scanner as well as two different MRI protocols. Field strength across all datasets was 3T.

**Table 2:** MRI acquisition parameters: imaging protocol parameters for all three datasets: RS1, RS2 and Clinical. The RS2 dataset was acquired at two different settings, so that 8/43 of the patients have different acquisition settings than the rest. The dataset is therefore split into two columns. All T1‑w images were acquired in 3D, all FLAIR images in 2D

Parameter	RS1	RS2		Clinical
**Patients (no.)**	50	35	8	53
**Scanner**	Philips Achieva dStream (Philips Healthcare, Best, The Netherlands)	Philips Achieva (Philips Healthcare, Best, The Netherlands)	Philips Achieva (Philips Healthcare, Best, The Netherlands)	Philips Achieva dStream (Philips Healthcare, Best, The Netherlands)
**FLAIR**				
*Voxel dim. (mm)*	0.59 × 0.59 × 3.84	0.49 × 0.49 × 3.85	0.93 × 0.93 × 3	0.59 × 0.59 × 3.84
*TR/TE (ms)*	11,000/125	11,000/125	10,000/80	11,000/125
*TI (ms)*	2800	2800	2600	2800
*Matrix size (voxels)*	384 × 384 × 35	512 × 512 × 35	256 × 256 × 44	384 × 384 × 35
*Flip angle (°)*	90	90	90	90
**T1**				
*Voxel dim. (mm*^*3*^*)*	0.7 × 0.7 × 0.7	1 × 1 × 1	0.97 × 1 × 0.97	–
*TR/TE (ms)*	11/5	8.15/3.73	8.06/3.69	–
*Matrix size (voxels)*	384 × 257 × 384	240 × 160 × 240	256 × 260 × 256	–
*Flip angle (°)*	8	8	8	–

*TR* repetition time, *TE* echo time, *TI* inversion time, *FLAIR* fluid attenuated inversion recovery

### Data Security

All patient specific data were handled in compliance with the Danish Data Protection Agency Act no. 502; thus, all patient data were completely anonymized. RS1 and RS2 were approved by the Ethics Committee of Copenhagen County according to the standards of The National Committee on Health Research Ethics and The Danish Data Protection Agency. All experiments were conducted in accordance with the Declaration of Helsinki of 1975 and all subjects gave written informed consent.

### Automatic Segmentation Model

Initially we implemented a state-of-the-art segmentation model, developed by Li et al. [22], which was declared the winner of the Medical Image Computing and Computer Assisted Intervention (MICCAI) White Matter Hyperintensity Segmentation Challenge [23]. The model was originally trained on the publicly available WMH dataset from the challenge, which was acquired on three separate scanners [23]. The model takes either a 2-channel input of 2D axial slices of T1‑w and T2‑w FLAIR MRI or just a 1-channel input of only T2‑w FLAIR. The output is a binary 2D lesion mask for each slice. In short, the model is a standard U‑net [24] with four down-sampling and four up-sampling blocks and skip connections in-between the two paths. Each block is comprised of two convolutional layers followed by either max-pooling or up-sampling and a rectified linear unit (ReLU) activation function. A schematic overview of the network structure is displayed in supplementary Table A.1. Preprocessing consists of Gaussian intensity normalization and standardization of the input image size by cropping or zero-padding. Data augmentation is applied through scaling, rotation, and shear mapping. For a thorough explanation of the methodology *see* [22].

### Implementation

We tested the 2‑channel variation of the model by Li et al. on our local validation dataset, which was resampled to the same resolution as the MICCAI data. Subsequently, we implemented the following changes in the network to make it applicable to our clinical set-up and to fine-tune the model to MS lesion delineation: 1) we extended the standardization size of input images (from 200 × 200 to 384 × 384 voxels) to accommodate the higher in-plane resolution of our data, 2) since we hypothesized an importance of having spatial information, we added the neighboring slice on either side of each relevant axial slice input thereby extending the network input to three channels, and 3) to reduce over-fitting when training, we added a drop-out layer after each convolutional layer in the U‑net, with a drop-out fraction of 20% and 4) the model was retrained on our local MS dataset in original resolution.

A one-sequence input was chosen as high-quality T1‑w images are not necessarily part of our clinical MS MRI protocol and broad applicability of the model was a priority.

### Training the Network

The network was retrained on the local RS1/RS2 dataset while applying transfer learning from the original model pretrained on MICCAI data. We used Adam as optimizer [25] with the dice similarity coefficient (DSC) as loss function and trained for 1000 epochs. The learning rate was set to 10^−5^ and batch size to 6. These values differ from the original training parameters by Li et al. [22]. The dataset of *n* = 93 was randomly split into training, validation, and testing, so that 73 patients were used for training the network, 10 patients (split 5/5 between RS1 and RS2) were used for validation and 10 patients (only RS1) were used for independent quantitative testing of the final segmentation model (Table 3). Input slices were randomly sampled during training.

**Table 3:** Distribution of patients from each dataset

Dataset	MICCAI	RS1	RS2	Clinical
*Training (pts)*	60	35	38	0
*Validation (pts)*	0	5	5	0
*Quantitative test (pts)*	0	10	0	0
*Qualitative test (pts)*	0	0	0	53

*Pts* patients, *MICCAI* Medical Image Computing and Computer Assisted Intervention

The four datasets were used for various aspects of model development and evaluation. Pretraining was performed using the MICCAI dataset, further training and evaluation was performed using RS1 and RS2 and an independent test using the clinical dataset.

### Manual Lesion Masks

All lesions in RS1 and RS2 were manually identified and delineated by one of three delineation experts following the guidelines from appendix A in [8] as part of their respective studies. Two of the experts had > 10 years of experience reading MS MRI, and the third was recently trained. All delineations were performed manually without using any computer assistance for guidance using the FSLeyes software [26]. A total of 3040 lesions were delineated in RS1 and RS2, averaging to approximately 32 lesions per patient. The manual delineations were used as reference masks during method development and training of our final model.

The reference masks of the test dataset were created in two steps to increase robustness. First, each of the two experienced delineators was asked to delineate the lesions individually, without any computer assistance. Second, each of them was presented with a dot-mask including the center of mass (CoM) marked in each lesion delineated by either one of the two experts or one of the automatic delineation tools, including our own. Each expert was then asked to delineate all lesions at the CoMs they agreed with. The second step was introduced to force the experts into deciding on all potential lesions, including areas that were difficult to differentiate from dirty-appearing white matter (DAWM) in an effort to increase interrater variability.

The time between step one and two was more than 6 months.

To comply with manual delineation standards, in which lesions with a maximum dimension below 3 mm are discarded [27], all lesions with a 3D volume below 10.7 mm^3^ were removed from all automatically and manually created lesion masks.

### Evaluation

#### Comparison to Other Available Segmentation Tools

We compared the performance of our adapted model to the performance of three available out of the box segmentation methods, to make sure the best model at hand was used for the clinical evaluation of automatic segmentation maps. The methods were ready for direct application but further trained on our local data in two instances. Each method was implemented and executed on the local test dataset, using default parameters as instructed by the respective authors. Our model only requires an input of T2‑w FLAIR images, and our dataset therefore does not contain all requested MRI sequences in all patients. In case of missing input sequences, the patient was omitted as explained below.

##### nicMSlesions

The nicMSlesions is a recent deep learning segmentation framework, based on a cascaded 3D CNN with T1‑w and T2‑w FLAIR as input [18, 20]. The model is validated and used for reference in several publications [17, 28, 29]. We tested the model in two variants: 1) using the pretrained model directly, 2) using the pretrained model for transfer learning while retraining on our local dataset. The dataset was limited to a subset of RS1/RS2 of 53 patients for training and 9 patients for testing, which included all 3 MRI sequences.

##### LST

The Lesion Segmentation Toolbox (LST) (version 3.0.0, www.statistical-modelling.de/lst.html) part of the software Statistical Parametric Mapping (SPM) includes two methods for lesion segmentation; an unsupervised lesion growth algorithm (LGA) [30] and a lesion prediction algorithm (LPA) based on logistic regression [31]. LGA requires T1‑w and T2‑w FLAIR, LPA requires only T2‑w FLAIR. The LGA dataset was therefore limited to a subset of RS1/RS2 of 9 patients for testing which included all 3 MRI sequences.

##### BIANCA

The Brain Intensity Abnormality Classification Algorithm (BIANCA) [32] is a k-nearest neighbor algorithm, which was fully trained on our local dataset, given T2‑w and T2‑w FLAIR modalities as input. The dataset was limited to a subset of RS1/RS2 of 72 patients for training and 9 patients for testing which included all 3 MRI sequences.

#### Quantitative Evaluation

Quantitative evaluation involved comparing the generated lesion maps with manual delineations, by computing three standard evaluation metrics described in Li et al. [22]. The metrics included assessment of lesion-delineation accuracy by calculation of the dice similarity coefficient (DSC), and measurements of lesion detection accuracy by F1 score and recall. The metrics were computed as follows:

$$\mathrm{DSC}=1-\frac{2\times \mathrm{TP}}{2\times \mathrm{TP}+\mathrm{FP}+\mathrm{FN}}$$

$$\textit{recall}=\frac{\mathrm{TP}}{\mathrm{TP}+\mathrm{FN}}$$

$$\mathrm{F1}=2\cdot\frac{\textit{precision}\cdot \textit{recall}}{\textit{precisiom}+\textit{recall}}, \quad\textit{with precision}=\frac{\mathrm{TP}}{\mathrm{TP}+\mathrm{FP}}$$

where TP, FP and FN are the number of true positives, false positives and false negatives, respectively. The first metric was calculated at voxel-level, while the latter two were calculated at lesion-level. A lesion was counted as a true positive if one voxel of a lesion in the generated mask is also part of a lesion in the reference mask.

Quantitative evaluation was performed on both the original model by Li et al., our adaptation of the model and the three public segmentation tools for comparison. We then investigated delineated volume differences between our model and the expert delineators by comparing total delineated volume of each patient. To compare the difference in delineated lesion volume between our model and the expert delineations with the difference between the two manual expert masks, we created a union mask. The union mask was created by merging the two manual delineations, while accepting all voxels delineated by both or just one of the experts. We then created two Bland-Altman plots: one presenting the difference between the two experts and one presenting the difference between our model and the union mask.

#### Qualitative Evaluation

To get an estimate of the value of the generated lesion masks by clinical specialists, we performed a qualitative clinical evaluation of our model delineations. We presented 2 experienced delineators with 53 generated masks from a local clinical dataset, displayed together with their corresponding MRI. Each patient and corresponding mask was displayed twice in a blinded, random fashion. The two experts compared the lesion masks to the original MRI and noted the number of false positive and false negative lesions for each mask. The mask was then categorized as either acceptable or unacceptable. The criteria for the two categories were designed by the two experts from their assessment of how many errors are expected due to delineation disagreements:

- Acceptable: more than 90% of the total amount of delineated lesions must be true positive, furthermore more than 90% of all true T2 lesions must be delineated.
- Unacceptable: more than 10% of all delineated lesions are false positive and/or more than 10% of all true T2 lesions are false negatives.

## Results

### Quantitative Evaluation

**Fig. 1:**
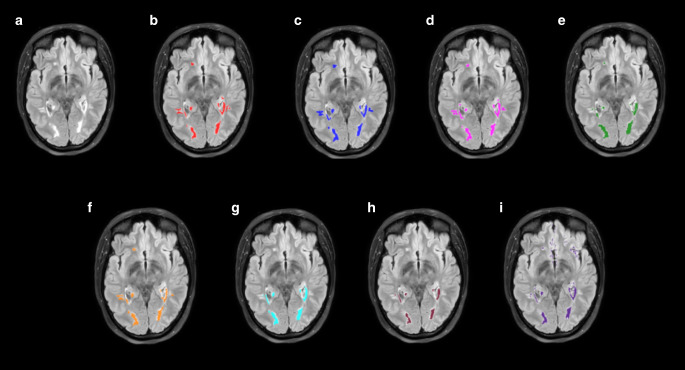
Example lesion delineations of all tested models including our implementation on an axial T2‑w FLAIR slice from a single patient. **a** T2‑w FLAIR, **b** expert 1, **c** adapted model (ours), **d** original model by Li et al., **e** nicMSlesions baseline, **f** nicMSlesions retrained, **g** LST-LGA, **h** LST_LPA, **i** BIANCA

**Fig. 2:**
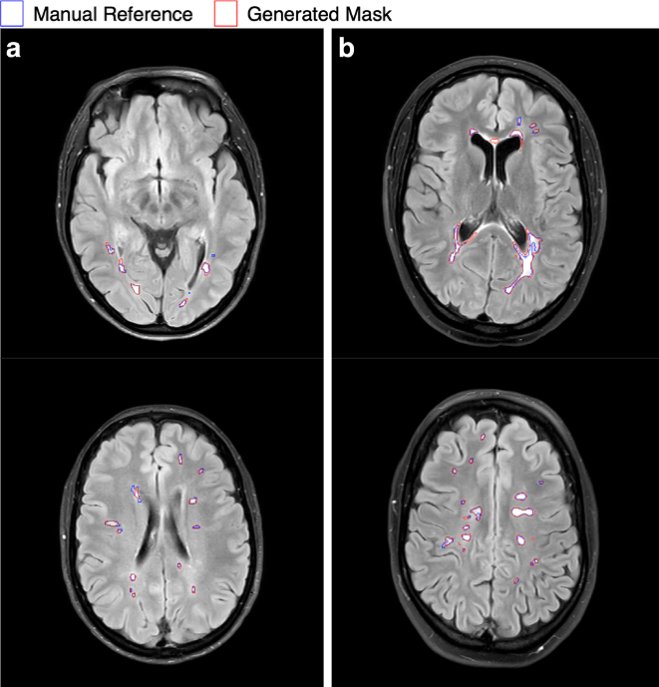
Two axial slices of T2‑w FLAIR images of two representative patients A and B. Delineations by our adapted segmentation model and clinical expert 1 are marked in red and blue, respectively. A good correspondence between the generated masks and manual reference masks is observed

The lowest mean dice scores were obtained by the three machine learning-based models BIANCA, LST-LGA and LST-LPA as well as the out-of-the-box implementation of the deep learning-based nicMSlesions. nicMSlesions achieved a large increase in mean DSC when retrained on part of our local dataset, rising from 0.41 to the second-best delineation score of 0.55. Similarly, the U‑net originally proposed by Li et al. increased performance from DSC 0.52 to 0.62 following our adaptation and retraining on our local dataset, outperforming the other models. The two retrained deep learning models also outperformed their out-of-the-box implementations when measured with F1 score and recall, with our adapted model achieving the highest mean performance metrics (Tab. 4). Examples of segmentation performance from each tested model compared to expert 1 are displayed in Fig. 1. Our adapted model exceeded interrater performance between the two delineators in the three categories (DSC: 0.57, F1: 0.68, recall: 0.58). Examples of the adapted model delineations compared to expert 1 are displayed in Fig. 2.

**Table 4:** The presented metrics were averaged across the two manual delineation masks and all 10 test patients. Standard deviation is presented in brackets. The best performance for each metric is highlighted in italics

Method	DSC	F1-score	Recall
BIANCA	0.34 (0.18)	0.30 (0.13)	0.56 (0.14)
LST-LGA	0.38 (0.13)	0.34 (0.10)	0.27 (0.18)
LST-LPA	0.42 (0.14)	0.39 (0.10)	0.32 (0.16)
nicMSlesions—baseline only	0.44 (0.12)	0.50 (0.15)	0.52 (0.16)
nicMSlesions—retrained on all patients	0.55 (0.12)	0.68 (0.19)	0.74 (0.11)
Original U‑net by Li et al	0.52 (0.12)	0.68 (0.15)	0.61 (0.14)
Adapted U‑net (ours)	*0.62 (0.12)*	*0.71 (0.18)*	*0.77 (0.10)*

*DSC* dice similarity coefficient, *BIANCA* Brain Intensity Abnormality Classification Algorithm, *LST-LGA* Lesion Segmentation Toolbox-lesion growth algorithm, *LST-LPA* Lesion Segmentation toolbox-lesion prediction algorithm

**Fig. 3:**
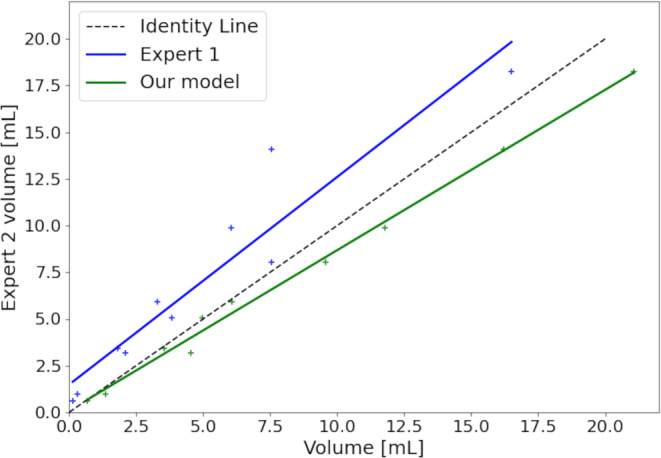
Delineated lesion volume per patient estimated by our model and expert 1 versus expert 2

We compared the total detected lesion volume per patient by our model against that of the two delineators. The results are presented in Fig. 3. The total lesion volume correlated to a high degree with the second expert delineator (*R*^2^ = 0.994), and to a lesser degree the first expert delineator (*R*^2^ = 0.899). The correlation between the two delineators was *R*^2^ = 0.899.

**Fig. 4:**
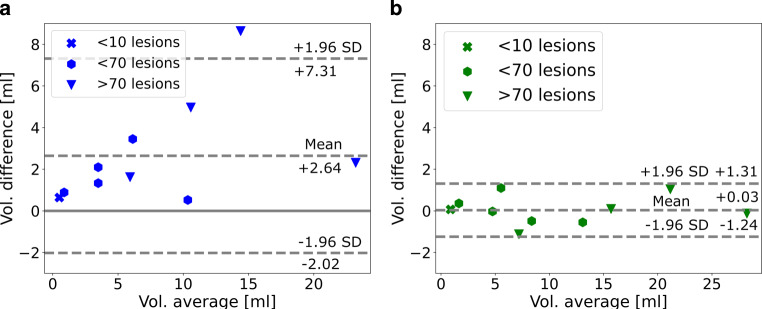
Bland-Altman plot of delineated lesion volume per patient of our adapted model and a union mask of the two delineators. **a** Expert 2 vs. expert 1. **b** Model vs. union mask (Expert1, Expert2)

Fig. 4 presents a Bland-Altman plot of the difference in delineated volume between the two experts as well as a plot of the difference between our adapted model and a union mask of the two delineators. The difference between our adapted model and the union mask is well in between the confidence interval of the interrater difference.

### Qualitative Evaluation

**Fig. 5:**
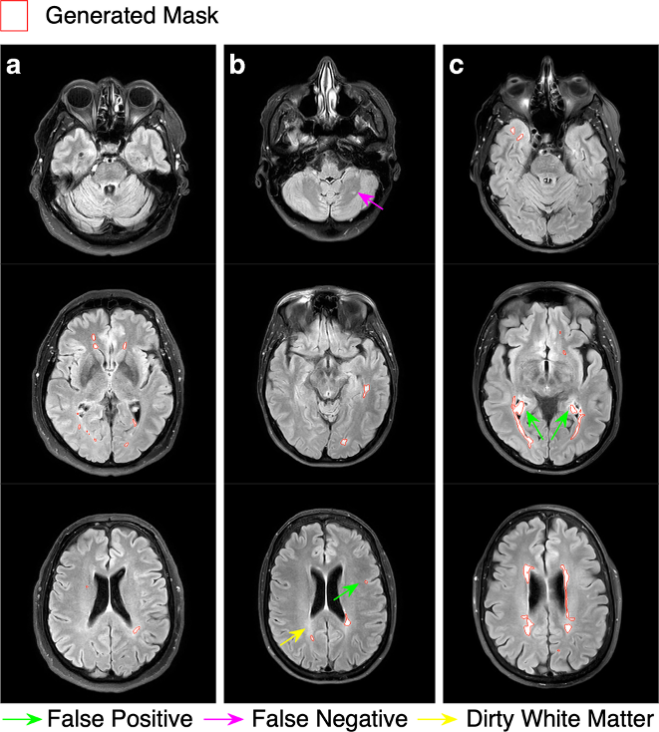
Three examples of masks evaluated by the clinical delineators. **a** Was rated as a perfect delineation mask. **b** Was rated as an acceptable delineation mask with some imperfections. These include a false negative lesion in the cerebellum marked by a purple arrow, a small false positive lesion in cortex marked with green and an area of DAWM, which is marked with yellow. **c** Was rated unacceptable. In the second slice large false positive delineations are drawn in plexus choroideus

The clinical evaluation of our produced lesion maps on our local clinical dataset resulted in 95.3% acceptable delineations and 4.7% unacceptable delineations as assessed by expert 1, as well as 93.4% acceptable and 6.6% unacceptable delineations as assessed by expert 2. This corresponds to the experts on average accepting 50 out of 53 segmentation masks. Slices of one of the rejected masks and two accepted are shown in Fig. 5.

## Discussion

In this study we implemented an automatic segmentation model with proven state-of-the-art performance on public challenge datasets and adapted it to our local MS dataset by updating the network structure and parameters, followed by a retraining to local data while transfer learning the original network parameters. The adapted automatic segmentation model produced lesion segmentation masks with a 94% acceptance rate in a clinical evaluation on our local dataset, suggesting a high possibility for implementation of automatically generated lesion masks in our clinical workflow. The model obtained segmentation scores above the interrater variability of two expert delineators, underlining the need for an automatic delineation procedure.

The out-of-the-box application of the model by Li et al., pretrained on the publicly available MICCAI, dataset achieved a DSC (0.52) within the previously reported range [9, 12, 17, 33], and outperformed the other out-of-the-box methods when evaluated on our dataset. After adapting the model we saw a significant increase in performance, achieving the highest DSC (0.62) across all evaluated methods, well above the interrater variability DSC (0.55), surpassing a frequently used segmentation framework [20]. These results confirmed previous findings [19, 20], highlighting the need for model adaptation to local data when implementing a pretrained segmentation method.

The quantitative evaluation is based on a test dataset of 10 subjects from a single scanner with a heterogenic number of MS lesions, ranging from 2 to 121 as counted by expert 1. The heterogeneity of the dataset appears to influence the metrics, as the lesion-wise metrics are relative to the number of total lesions, and results in a relatively high variance between the 10 test patients, which complicates generalization of the averaged metric scores to a larger cohort. Our implemented model generally segmented more and larger areas than the manual delineators, which resulted in a high recall score (most of the manual lesions were identified by the model) but lower f1 score (overall segmentation performance). False positive segmentation can be problematic during monitoring of MS patients, as new or enlarged lesions on an MRI can result in a change in treatment plans. Radiologists should therefore be aware of false positive segmentations, when examining the masks in the clinical setting. In both the lesion volume plot and the metric scores our implemented model had a higher agreement with expert 2 than expert 1, who generally delineated smaller and fewer volumes. Interestingly, the lesion volume analysis showed that the delineated volume by expert 2 was closer to the automatic delineations than to expert 1, which indicates that part of the disagreement between expert 1 and the model can be attributed to differences in delineation style. When inspecting the Bland-Altman plots of total volume per patient, the differences between our adapted model and the union mask are well within the confidence interval of the two experts, indicating that the automatically delineated lesion volume is within interrater variability.

We found that a large part of the false positive delineations of the model were areas of DAWM, which is common in clinical MS data. DAWM are hyperintense areas of white matter which have an intensity between that of normal-appearing white matter and lesions [34]. DAWM can be difficult to separate from the MS lesions due to their diffuse boundaries, which increases the complexity of the segmentation task. They further complicate evaluation against a manual reference, since differentiation between lesions and DAWM is difficult to standardize. To the best of our knowledge, at the time of this paper, there is no universally adopted method to tackle DAWM in lesion segmentation [35] An approach for future research could be to either mask out areas of DAWM or to consistently include these areas in manual delineation, and to include a clear statement on which approach was selected.

Since the original model by Li et al. was developed to delineate WMH of presumed vascular origin and not MS lesions, the model might be biased towards delineating WMH as well as DAWM, which can be located in the same areas as vascular WMH; however, when delineating WM lesions in MS patients, it is common consensus to also include subcortical lesions (above a certain size and shape threshold), disregarding the fact that some of these lesions might potentially be of vascular age-related origin. For this reason, we find it appropriate that the algorithm is able to detect this lesion type as well.

We sought to adapt the model to delineation of MS lesions instead of general WMH by retraining the model on MS data and optimizing model parameters. The increase in performance between our adapted model and the model by Li et al. indicates that the model has been optimized for MS lesion segmentation; however, we have not tested the models’ ability to differentiate between, e.g. vascular and MS lesions.

There is a discrepancy between our model’s recall score at 77% in the limited test dataset and the fact that 94% (average) of the clinical masks were rated acceptable in clinical practice when an acceptable mask should allow a maximum of 10% incorrect delineations. This discrepancy could be partially due to differences in reference. In the quantitative evaluation, we created the dot-masks from all delineations to force the experts to classify any possible lesion. In the qualitative evaluation, only delineations from our model were presented. This difference in set-up might bias the delineators into delineating more lesions in the former than in the latter. The interrater recall score of 0.50 also points to a large uncertainty regarding lesion delineation in the small test dataset.

The aim of this study was to implement and evaluate a model to tackle delineation of MS patients routinely examined at our institution. Two patient cohorts were included in the dataset: optic neuritis (ON) and recurrent remitting MS (RRMS). Both cohorts are in the early stages of MS, and the dataset could therefore be biased towards low lesion counts. The RRMS cohort made up the largest part of the training data and the entire part of both testing datasets. Since approximately 85% of all MS patients are RRMS patients, robust delineation of these patients in a clinical setting is of great importance [36]; however, because of the relative homogeneity of the dataset, the adapted and retrained model is not generalizable to other more diverse patient cohorts, e.g. patients examined on other scanners or diagnosed with progressive forms of MS. This would, again, require retraining of the model.

The high acceptance rate in the clinical evaluation (94%) suggests that our implemented model could assist in both clinical routines today and in future applications. As lesion delineation is not part of clinical routine today, current application of the model would be to lighten the lesion registration burden for radiologists during diagnosis and monitoring of MS patients. Through delineation the model can produce default lesion registration immediately after MRI acquisition, which can be subsequently edited as needed by the radiologist. Although unusual cases might require a more thorough editing, in most cases (94%) the use of the automatic segmentation tool will considerably speed up the segmentation process, while also decreasing registration variance. A future application could be in implementation of new research, such as MS prognostics and early stratification [6, 37], which require either full lesion delineation masks or total lesion load as an input.

## Conclusion

In this study we have implemented an artificial intelligence (AI) state-of-the-art deep learning model for automatic WMH delineation in MS and adapted it for optimal performance on our local single-center MS dataset. The model was quantitatively evaluated on a small test dataset and qualitatively evaluated on a larger clinical dataset, aimed to explore the clinical value of automatic MS lesion segmentation in our clinic.

Our adapted model obtained a segmentation performance exceeding both out-of-the-box lesion segmentation as well as manual interrater performance across most assessed metrics, underlining the need for automatic delineation models retrained on local data. A clinical evaluation performed by two experienced MS lesion delineators, resulted in an acceptance of 94% of the generated lesion masks on our local clinical dataset. The high clinical acceptance rate is a promising step towards implementation of automatic delineation models in our clinical routine, although special attention should be given to false positive delineations.

## Supplementary Information

Network architecture overview, Individual metrics scores of our model regarding each delineator and individual metric scores of our model for each test patient.
