# Supplementary material for: Assessment of Artificial Intelligence Automatic Multiple Sclerosis Lesion Delineation Tool for Clinical Use
Source: Clin Neuroradiol. 2021 Sep 20;32(3):643–53. doi: 10.1007/s00062-021-01089-z (PMC9424132; doi:10.1007/s00062-021-01089-z)
Supplement: Supplementary file 1 — Network architecture overview, Individual metrics scores of our model regarding each delineator and individual metric scores of our model for each test patient. [file 62_2021_1089_MOESM1_ESM.docx]

# Supplementary

# A. Network architecture

# **Table A.1, Network Architecture:** Schematic presentation of the original U-net architecture from Li et al. [22]. All convolutional layers had stride 1. The network had skip-connections between layers at same level in the encoding and decoding paths. Conv: convolution, ReLU: Rectified Linear Unit.

|  | **Layer** | **Parameters** | **Nr of feature maps** | **Activation** | **Output** |
| --- | --- | --- | --- | --- | --- |
|  | Input |  |  |  | 200x200 |
| **Encoding path** | Conv 1a | 5x5 | 64 | ReLU | 200x200 |
|  | Conv 1b | 5x5 | 64 | ReLU | 200x200 |
|  | Max-pool | 2x2, stride 2 |  |  | 100x100 |
|  | Conv 2a | 3x3 | 96 | ReLU | 100x100 |
|  | Conv 2b | 3x3 | 96 | ReLU | 100x100 |
|  | Max-pool | 2x2, stride 2 |  |  | 50x50 |
|  | Conv 3a | 3x3 | 128 | ReLU | 50x50 |
|  | Conv 3b | 3x3 | 128 | ReLU | 50x50 |
|  | Max-pool | 2x2, stride 2 |  |  | 25x25 |
|  | Conv 4a | 3x3 | 256 | ReLU | 25x25 |
|  | Conv 4b | 3x3 | 256 | ReLU | 25x25 |
|  | Max-pool | 2x2, stride 2 |  |  | 25x25 |
| **Base block** | Conv 5a | 3x3 | 512 | ReLU | 12x12 |
|  | Conv 5b | 3x3 | 512 | ReLU | 12x12 |
| **Decoding path** | Up-conv | 2x2 |  |  | 24x24 |
|  | Conv 6a | 3x3 | 256 | ReLU | 24x24 |
|  | Conv 6b | 3x3 | 256 | ReLU | 24x24 |
|  | Up-conv | 2x2 |  |  | 48x48 |
|  | Conv 7a | 3x3 | 128 | ReLU | 48x48 |
|  | Conv 7b | 3x3 | 128 | ReLU | 48x48 |
|  | Up-conv | 2x2 |  |  | 96x96 |
|  | Conv 8a | 3x3 | 96 | ReLU | 96x96 |
|  | Conv 8b | 3x3 | 96 | ReLU | 96x96 |
|  | Up-conv | 2x2 |  |  | 192x192 |
|  | Conv 9a | 3x3 | 64 | ReLU | 192x192 |
|  | Conv 9b | 3x3 | 64 | ReLU | 192x192 |
|  | Zero - padding |  |  |  | 200x200 |
| **Output** | Conv 10 | 1x1 | 1 | Sigmoid | 200x200 |

# Individual metric scores for our model for each delineator

# **Table B.1, Quantitative results, delineator 1:** Average metrics across the 10 test patients when compared to delineator 1. LST: Lesion Segmentation Tool, LGA: Lesion Growth Algorithm, LPA: Lesion Prediction Algorithm, DSC: Dice Similarity Coefficient.

| **Method** | **DSC** | **F1** | **Recall** |
| --- | --- | --- | --- |
| BIANCA | 0.35 (0.18) | 0.30 (0.14) | 0.64 (0.17) |
| LST-LGA | 0.44 (0.12) | 0.39 (0.11) | 0.34 (0.25) |
| LST-LPA | 0.49 (0.18) | 0.44 (0.12) | 0.40 (0.23) |
| nicMSlesions – baseline only | 0.46 (0.13) | 0.55 (0.17) | 0.64 (0.21) |
| nicMSlesions – retrained on all patients | 0.54 (0.14) | 0.72 (0.20) | 0.85 (0.09) |
| Original U-net by Li et al | 0.52 (0.15) | 0.72 (0.17) | 0.68 (0.16) |
| Adapted U-net (ours) | 0.58 (0.14) | 0.73 (0.12) | 0.90 (0.07) |

# **Table B.2, Quantitative results, delineator 2:** Average metrics across the 10 test patients when compared to delineator 2. LST: Lesion Segmentation Tool, LGA: Lesion Growth Algorithm, LPA: Lesion Prediction Algorithm, DSC: Dice Similarity Coefficient.

| **Method** | **DSC** | **F1** | **Recall** |
| --- | --- | --- | --- |
| BIANCA | 0.34 (0.17) | 0.30 (0.13) | 0.48 (0.10) |
| LST-LGA | 0.31 (0.14) | 0.28 (0.09) | 0.20 (0.10) |
| LST-LPA | 0.36 (0.15) | 0.34 (0.09) | 0.24 (0.09) |
| nicMSlesions – baseline only | 0.41 (0.11) | 0.45 (0.13) | 0.41 (0.11) |
| nicMSlesions – retrained on all patients | 0.55 (0.10) | 0.65 (0.17) | 0.6259 (0.13) |
| Original U-net by Li et al | 0.52 (0.09) | 0.65 (0.12) | 0.53 (0.13) |
| Adapted U-net (ours) | 0.66 (0.10) | 0.69 (0.16) | 0.63 (0.12) |

# Metric scores of our adapted model for each patient

# Individual patient metrics from the 6 tested models can be found in S2.

# **Table C.1, Individual patient metrics:** Metric scores for each of the ten test patients of the adapted segmentation model with respect to the two delineation experts. DSC: Dice similarity coefficient. AVD: Average volume difference.

| **Metric scores of our adapted model for each patient against expert 1** | | | | | | | | | | |
| --- | --- | --- | --- | --- | --- | --- | --- | --- | --- | --- |
| **Patient** | **1** | **2** | **3** | **4** | **5** | **6** | **7** | **8** | **9** | **10** |
| DSC | 0.62 | 0.54 | 0.58 | 0.33 | 0.34 | 0.56 | 0.63 | 0.74 | 0.69 | 0.78 |
| F1 | 0.90 | 0.75 | 0.81 | 0.43 | 0.29 | 0.76, | 0.79 | 0.84 | 0.78 | 0.96 |
| Recall | 0.96 | 0.79 | 0.96 | 0.78 | 1.0 | 0.86 | 0.93 | 0.96 | 0.89 | 0.87 |
| Nr of lesions by expert 1 | 45 | 121 | 110 | 9 | 2 | 52 | 32 | 56 | 70 | 86 |

| **Metric scores of our adapted model for each patient against expert 2** | | | | | | | | | | |
| --- | --- | --- | --- | --- | --- | --- | --- | --- | --- | --- |
| **Patient** | **1** | **2** | **3** | **4** | **5** | **6** | **7** | **8** | **9** | **10** |
| DSC | 0.70 | 0.60 | 0.68 | 0.46 | 0.56 | 0.63 | 0.67 | 0.76 | 0.73 | 0.81 |
| F1 | 0.80 | 0.712 | 0.77 | 0.38 | 0.42 | 0.68 | 0.76 | 0.72 | 0.81 | 0.89 |
| Recall | 0.64 | 0.62 | 0.72 | 0.36 | 0.57 | 0.57 | 0.60 | 0.64 | 0.79 | 0.81 |
| Nr of lesions by expert 2 | 71 | 155 | 152 | 19 | 7 | 78 | 56 | 70 | 88 | 83 |
